# Country Income Is Only One of the Tiles: The Global Journey of Antimicrobial Resistance among Humans, Animals, and Environment

**DOI:** 10.3390/antibiotics9080473

**Published:** 2020-08-01

**Authors:** Angela Pieri, Richard Aschbacher, Giada Fasani, Jole Mariella, Lorenzo Brusetti, Elisabetta Pagani, Massimo Sartelli, Leonardo Pagani

**Affiliations:** 1 Infectious Diseases Unit; Bolzano Central Hospital, 39100 Bolzano, Italy; angela.pieri@sabes.it; 2 Antimicrobial Stewardship Program; Bolzano Central Hospital, 39100 Bolzano, Italy; richard.aschbacher@sabes.it (R.A.); giadafasani@hotmail.com (G.F.); elisabetta.pagani@sabes.it (E.P.); 3 Department of Veterinary Medical Sciences (DIMEVET), University of Bologna, 40126 Bologna; Italy; jole.mariella2@unibo.it; 4 Faculty of Science and Technology; Free University of Bolzano, 39100 Bolzano, Italy; lorenzo.brusetti@unibz.it; 5 Division of General Surgery, Macerata Hospital, 62100 Macerata; Italy; massimosartelli@gmail.com

**Keywords:** antimicrobial resistance, One Health, environment, humans, animals, antibiotic resistance genes, water, antimicrobial stewardship

## Abstract

Antimicrobial resistance (AMR) is one of the most complex global health challenges today: decades of overuse and misuse in human medicine, animal health, agriculture, and dispersion into the environment have produced the dire consequence of infections to become progressively untreatable. Infection control and prevention (IPC) procedures, the reduction of overuse, and the misuse of antimicrobials in human and veterinary medicine are the cornerstones required to prevent the spreading of resistant bacteria. Purified drinking water and strongly improved sanitation even in remote areas would prevent the pollution from inadequate treatment of industrial, residential, and farm waste, as all these situations are expanding the resistome in the environment. The One Health concept addresses the interconnected relationships between human, animal, and environmental health as a whole: several countries and international agencies have now included a One Health Approach within their action plans to address AMR. Improved antimicrobial usage, coupled with regulation and policy, as well as integrated surveillance, infection control and prevention, along with antimicrobial stewardship, sanitation, and animal husbandry should all be integrated parts of any new action plan targeted to tackle AMR on the Earth. Since AMR is found in bacteria from humans, animals, and in the environment, we briefly summarize herein the current concepts of One Health as a global challenge to enable the continued use of antibiotics.

## 1. Introduction

Antibiotics are the foundation of modern medicine: they are the reason for the survival of immunocompromised patients and transplant recipients, they support trauma care, as well as implants or prostheses; the whole medical world relies on the possibility to treat infections: without safe and effective antibiotics, indeed, the whole edifice of modern medicine itself will crumble because of the spread of antimicrobial resistance (AMR) [1].

Several interconnected human, animal, and environmental habitats can contribute to the emergence, evolution, and spread of AMR, and it is essential to recognize that the health of people is tightly connected to the health of animals, that diseases are transmitted from humans to animals and vice versa, and that AMR must therefore be tackled in both. Such a perspective also encompasses the environment, which is clearly tied to humans and animals and likewise a potential source of new resistant microorganisms. In addition, antibiotics are often used for nonmedical uses, such as beekeeping, ethanol production, horticulture, in antifouling paints to prevent barnacle build-up on boat hulls, and food preservation [2]. Unfortunately, while society now takes antibiotics for granted, it has been shown that knowledge and attitudes regarding antibiotic use can be, and must be, improved and that improved knowledge may help reduce the misconceptions and misguided expectations contributing to inappropriate antibiotic use [3,4].

The defined “One Health” approach is essential for developing comprehensive and integrative measures to address AMR. This approach should include the surveillance of microbes in humans, animals, and environments to better understand AMR, and to develop effective preventive and control strategies [5]. One Health is defined as a concept of “designing and implementing programs, policies, legislation and research in which multiple sectors communicate and work together to achieve better public health outcomes”. Likewise, it needs to involve the “collaborative effort of multiple health science professions, together with their related disciplines and institutions—working locally, nationally, and globally—to attain optimal health for people, domestic animals, wildlife, plants, and [the] environment” [6].

In this review, we summarize the current knowledge about the three parts that make up our world from the globally expanding threat of AMR.

## 2. AMR in Humans

AMR is one of the greatest health challenges of the 21st century [1,7,8,9,10,11,12,13]. On the one side, the overuse and misuse of antimicrobials are certainly among the major drivers for the development of AMR globally, but on the other side, a lack of laboratory resources or skills to identify the organism and its antimicrobial susceptibility, with unreliable or absent surveillance data on AMR, a lack of perception or accountability of the threat, and a lack of treatment guidelines place certain health care facilities at risk for sustaining the development of AMR. Inadequate compliance to infection control and prevention (IPC) measures in health care facilities and poor hygiene and sanitation in communities worsen the spread of infections and increase the use of antimicrobial agents. 

Besides, patient and public expectation and pressure to prescribe antibiotics, or any situation that allows for financial benefit from the supply of medicines, such as unregulated over-the-counter availability and use, can drive inappropriate antimicrobial prescribing. Indeed, another major threat that fosters the spread of AMR and endangers the patients’ safety is drug counterfeit. As a consequence of globalization, a criminal market of drug without standard manufacture requirements has flourished, and even antimicrobials were—and indeed are—not exempt from this phenomenon. Warnings and alerts have arisen in the last years all around the world [14,15,16,17], and different experiences and investigations have clearly shown the impact of counterfeit drugs not only on morbidity and mortality in low- and middle-income countries, but also on enhancing the spread of AMR [16,18,19,20,21]. 

AMR is widespread in Gram-positive and Gram-negative bacteria, and the following resistance phenotypes have a significant clinical public health impact: methicillin-resistant *Staphylococcus aureus* (MRSA), vancomycin-resistant enterococci (VRE), extended-spectrum β-lactamase (ESBL), and high-level AmpC producing *Enterobacteriaceae* and carbapenemase producing *Enterobacteriaceae, Pseudomonas aeruginosa*, and *Acinetobacter baumannii* [22]. Generally, the above-mentioned phenotypes are associated, besides β-lactam antibiotics, with resistance to various other antibiotic classes, and give rise to multi-drug resistance (MDR). Moreover, many resistance genes are located on mobile genetic elements that are able to move within or between DNA molecules, which include transposons and gene cassettes/integrons, or are able to horizontally transfer between bacterial cells, such as plasmids and integrative conjugative elements [23]. AMR mechanisms encoded by genes have a far greater impact on transfer than mutations. Depending on how the resistance mechanism is transferred, the power of dissemination is different: by vertical transfer of the resistance gene (as mostly happens for *S. aureus* and Gram-positive pathogens), it will be transmitted to the next generations. In the case of horizontal transfer (mostly in *Enterobacterales* and Gram-negative pathogens), the resistance gene moves to neighboring bacteria, and therefore, the spread of resistance can be even greater [24,25].

Healthcare institutions such as hospitals, nursing homes, and rehabilitation facilities are hotbeds for MDR bacteria, but some MDR organisms have become quite prevalent causes of community-acquired infections (e.g., ESBL-producing *E. coli*, community-acquired-MRSA); the spread of MDR bacteria into the community is a crucial development, and it is associated with increased morbidity, mortality, healthcare costs, and once again, antibiotic overuse [26]. 

However, humans themselves may act as a potential reservoir and spread AMR: antibiotics in the early stages of life are capable of definitively altering the microbiome with the selection of resistance in some commensal strains, then triggering the spread of such mechanisms to others [8,27]; moreover, extensive gene transfer has been shown to occur among bacteria in the human colon, not only within one genus (*Bacteroides*), but also among *Bacteroides* species and residing Gram-positive bacteria [28].

The threats posed by AMR are of increasing concern even in low- and middle-income countries (LMICs), as their rates of antibiotic use increase. An understanding of the burden of resistance is rather lacking in LMICs, particularly for MDR pathogens. 

AMR spread affects not only the national budgets or gross domestic product (GDP) of countries, but also the attributable mortality or related disabilities. Cassini et al. [29], through a population-level modeling analysis, estimated the burden of infections caused by antibiotic-resistant bacteria in countries of the EU and European Economic Area (EEA) in 2015, which was measured in the number of cases, attributable deaths, and disability-adjusted life-years (DALYs). To understand the ominous burden and the practical meaning of such results, it may be sufficient to figure that the burden of infections by antibiotic-resistant pathogens is similar to the cumulative burden of tuberculosis, influenza, and HIV [29].

One important conclusion that can be drawn from this study is that most of the estimated burden was in hospitals or other healthcare settings, thus suggesting the urgent need to address AMR as a patient safety issue. 

## 3. AMR in Animals

Antimicrobial classes that are used in human and animal health encompass the same or very similar molecules to a large extent, and this can clearly drive the transmission of resistance between animals and people, either directly or via the environment. Antimicrobials used in animal farming are often administered in sub-therapeutic doses and for prolonged periods, thus framing ideal conditions for bacteria to fix genes that confer resistance, and contributing fundamentally to the global AMR crisis. These genes can subsequently be transferred to human-adapted pathogens or to human gut microbiota especially through contaminated food at origin, an unsafe food chain, or the environment. Moreover, they support ideal conditions for the amplification of genes that may have arisen in people or the environment [6]. 

The relationship between health and AMR in the food chain encompasses both the pathogenic and non-pathogenic microorganisms, as both can have serious consequences [30]. There are two major concerns about human health: (1) the safety of food chain, which is the issue of preventing food from being contaminated by pathogenic strains, and (2) the emergence and spread of AMR in livestock, intensive poultry farming, or aquaculture, with the consequent transfer of AMR sequences or genes to consumers. This is mostly related to the massive use of antibiotics in food animals worldwide [31,32], but also to mobile genetic elements related to AMR that may survive and circulate among different human and animal settings [33,34].

The first one is the potential unsafety of food linked to contaminated water, preparation, or poor hygiene [35,36,37,38]. Vegetables and mussels contaminated with Gram-negative carriers of ESBL or KPC-3 carbapenemase in retail markets in North Africa, or imported seafood and raw dog food with the presence of *mcr-1* positive *E. coli* isolates in Norway, have been recently reported, thus highlighting new potential pathways to transfer AMR genes to “low prevalence” countries [39,40,41,42,43]. 

Antimicrobials have been and are still widely used for disease prevention and growth promotion in food animals. Expanding human population ever demands more animal-based protein, which in turn leads to more industrialized methods of food animal production, including sub-therapeutic antibiotics for growth promotion and disease prevention [44,45,46,47]. This means that animal bacteria may become resistant under selective pressure; then, bacteria travel from farms to stores, and then they may cause hard-to-treat infections in the final consumer as a consequence [48,49,50,51]. The relationship between massive use of antibiotics to treat or prevent illnesses or for growth purposes and the consequent emergence of several AMR mechanisms in food animals followed by spread into the environment has been clearly highlighted in several reports [52,53,54,55,56,57,58,59,60,61]. Very recently, the first cases of linezolid-resistant coagulase-negative staphylococci recovered in healthy turkeys in Egypt have been reported, as did the massive use of antibiotics in African countries in food-producing animals [62,63].

The high proportion of poor-quality veterinary medicine for therapeutic use in livestock exacerbates the problem of antibiotic overuse or misuse, particularly in LMICs [64]. As seen for human drugs, poor-quality medicines that provide sub-therapeutic doses of active pharmaceutical ingredients, whether due to inadequate amounts of pharmaceutical, ineffective release, the presence of impurities, or the degradation of compounds, are believed to contribute to AMR by exposing microbes to a level of antibiotic that will not effectively kill the whole microbial population [64].

Intensive farming is also a stress factor for animals due to crowding and a lack of hygiene, causing illnesses and increased shedding. Antibiotics consumed by animals can be excreted in urine and feces into the surrounding environment, potentially inducing or selecting for the development and maintenance of antibiotic resistance genes (ARGs) into the environment [65,66,67]. Infection control and prevention procedures and sanitation are concepts still not fully developed in animal farming both in very intensive farming in Western countries and in poor-resource or developing countries, and large efforts are requested at the national level in every country to tackle the spread of AMR into the environment. 

In 2014, China produced over 45 million metric tons of fish, crustaceans, and mollusks by aquaculture with more than 50% of this production exported [68]. The heavy use of colistin and other antimicrobials in this industry in China may have generated plasmid-mediated colistin resistance genes *mcr*-1 and *mcr*-2 through the facilitation of the capture and dissemination of potential colistin resistance genes from aquatic bacteria, such as *Aeromonas* and *Shewanella*, which can be naturally resistant to colistin [68]. This hypothesis seems to be confirmed by a recent work from Chinese researchers, who explored the molecular characteristics and relationships of *mcr*-1 positive *Escherichia coli* through Whole-Genome Sequencing (WGS), concluding that these strains were highly prevalent in the aquaculture supply chain and resistant to most antibiotics, and that *mcr*-1 could be transferred to humans via the aquatic food chain [69]. Likewise, Zhang et al. analyzed a total of 860 aquatic samples from three types of retail aquatic products collected from 39 major cities in China from 2011 to 2016 focusing on the main characteristics of *Bacillus cereus*, and found 100% antibiotic resistance to rifampin and most β-lactams, thus highlighting the need for continuously monitoring AMR in *B. cereus* in aquatic products and controlling drug use in aquaculture [70].

The higher the amount of antibiotics used in animals, whatever the reason, the higher the anticipated AMR rates [49]. Moreover, a clear direct relationship between these facts has been demonstrated also in either sense: broad antibiotic restrictions in food animals decrease rates of resistance. When the glycopeptides avoparcin was banned across the European Union in the 1990s, the prevalence of vancomycin-resistant enterococci from both poultry and humans decreased [71]. However, the ban of antibiotics as growth supplements for farm animals cannot be the unique solution: developing antibiotic alternatives to combat the global increase in AMR and the successful agricultural use of various phytochemicals and their mode of action in major agricultural animals (poultry, swine, and ruminants), replacing antibiotics in more sustainable animal production, have been shown to be very promising [72].

The last aspect to be considered in the strict interplay between humans and animals about AMR is the relationship of humans with pets, companion animals, or animals living close to humans for work or recreational activities. Dogs are not only potential carriers of MRSA, but they can also be colonized by plasmid-mediated quinolone resistance genes or different ESBL-producing *Enterobacterales* [73,74,75]. 

Overall, these studies highlight also the importance of raising awareness in patients and healthcare workers: even if pets or companion animals may be very helpful for some “frail” patients for their social activities, nevertheless, they must be trained to optimal animal waste disposal and contact precautions, ideally by “trained trainers”; this would avoid patient colonization with MDR pathogens. 

## 4. AMR in the Environment

AMR develops in, and is maintained and transmitted across humans, animals, and the natural environment. In the natural environment, antibiotics are produced by microorganisms to better compete against other microbial competitors, or just as molecular signaling between cells [76]. Therefore, it is not surprising that natural environments present a transmission route and a reservoir for resistant microorganisms. Resistance is an ancient and naturally occurring phenomenon, and it can be linked to even other environmental factors besides antibiotics. For instance, it is the case of heavy metals: bacterial efflux pumps, located onto their cell membranes, may act against antibiotics [77]. Then, pollutants such as heavy metals, but also other pollutants such as quaternary ammonium compounds, antifouling agents, and detergents may also affect the frequencies of antibiotic resistance through linked selection, even at low concentrations [77,78,79].

Antibiotic-producing microorganisms can protect themselves from the toxic effect of the drug using different strategies: one of the most common involves the modification of the antibiotic’s target site. The soil bacterium *Streptomyces* sp. strain AM-2504 exerts a molecular mechanism to protect itself from the activity of dityromycin that can be reproduced in E. coli, which is one of the commonest commensals in humans [80]. 

Antibiotic resistance genes (ARGs) are ubiquitous in nature [81]: they can be found in high concentrations in clinical, industrial, and urban wastewater, as well in animal husbandry [82]; even trace amounts of sewage recovered from environmental fresh waters may support the diffusion of integrons and ARGs [83]. The aquatic environment is one of the most important reservoirs for the onset, maintenance, and spread of different AMR mechanisms, such as the horizontal transfer of virulence factors by enhanced rates of conjugation [84,85]. 

Moreover, these environments frequently contain very high levels of antibiotics and pharmaceuticals [86,87]: the impact of uncontrolled discharge of partially treated or untreated wastewater on the structure of bacterial communities and resistome of sediments collected in some Indian districts using shotgun metagenomics has been clearly investigated, and a wide array of carbapenemases such as NDM, VIM, KPC, OXA-48, and IMP, which are horizontally transferable ARGs, were detected [88]. Remarkably, the abundance of ARGs was 30-fold higher in river sediments within the city compared to upstream sites. In addition to ARGs, higher abundances of mobile genetic elements were found in city samples, as well as some biocide/metal resistance genes. *Acinetobacter* comprised up to 29% of the 16S rRNA reads, and a strong correlation was found between the abundance of *Acinetobacter* and the OXA-58 carbapenemase gene [88]. Other experiences from India confirmed these data, and integrated plans to protect water should clearly be implemented soon [89,90,91].

The counterpoint of any human activities potentially releasing whatever ARG in the environment is that MDR bacteria or even pathogens excreted or eliminated via effluents and sewage can re-contaminate humans and animals [92] even during recreational activities, and this must be taken into account when frail or immunosuppressed patients get in close contact with these environments [93,94,95]. 

Surprisingly, even remote environments or the sponge microbiota may host AMR genes or plasmids [84,96], and novel antibiotic resistance determinants have been also discovered in forest and grassland soil metagenomes [97], thus hosting diverse and novel resistance genes that may be harnessed by phylogenetically distinct bacteria and may act as a reservoir of functional resistance genes [98]. The occurrence of MDR bacteria in wildlife is clearly influenced by many and different factors that have not yet been fully understood [99,100]. Most research has focused on measuring concentrations of antibiotics and characterizing the abundance and diversity of ARGs and MDR bacteria in the environment, but there has been limited empirical research on whether humans are exposed to this, and whether exposure can lead to measurable impacts on human health [101].

Finally, it must be reminded that healthcare settings themselves may act as a potential reservoir for environmental pollution with MDR or even hospital-acquired outbreaks with MDR pathogens [102,103].

Therefore, integrated surveillance between human medicine, veterinary medicine, and the environment is of paramount importance to detect and track emerging threats over time [47,104]. AMR surveillance data can also inform the development of treatment guidelines. Three main synergistically acting surveillance levels can be prospected to improve health outcomes:

1. Local. Allow healthcare professionals to make better informed clinical decisions to ensure better patient outcomes.
2. National. Guide policy and ensure appropriate and timely public health interventions.
3. Global. Provide early warnings of emerging threats and data to identify and act on long-term trends.

Yet it has proven difficult to achieve these objectives on a global scale, and especially in LMICs, largely because current surveillance systems deliver data that are extremely variable in quality and quantity [104]. Nowadays, water is one of the most important targets and opportunities to track and investigate AMR from very different points of view [105,106]; Aarestrup and Woolhouse recently proposed a very promising plan for a global AMR surveillance system based on the quantification of AMR genes in sewage that will be especially helpful for community AMR surveillance, and it will provide an affordable surveillance option even in resource-poor settings or remote locations without microbiology laboratories [104]. 

However, an integration of different surveillance systems for animal and human health encompassing also environmental concerns is unfortunately still lacking among most of the countries: it has been clearly indicated that despite huge but often individual efforts, even the most relevant AMR action plans are partly deficient because they do not tackle all the potentially relevant pathways and drivers of AMR in the environment, and the science to inform policy is still lacking [107]; these gaps must be addressed without delay, and great, concerted efforts should be invested in this fundamental branch of One Health [108]. 

## 5. Conclusions

AMR is one of the most complex global health challenges today, and antibiotics are the foundation of modern medicine: not only the medical world, but also the planetary health rely on the possibility of treating infections: without safe and effective antibiotics, the “main building” of modern medicine itself will crumble, after decades of overuse and misuse in human medicine, animal health, agriculture, and dispersion into the environment [107]. 

The One Health concept stresses the ecological relationships between human, animal, and environmental health [109]: since AMR is found in bacteria from animals and humans, it is a problem that cannot be solved by looking at either in isolation: we should start acting jointly to protect the whole world and the planetary health from the threat of AMR in every setting of our life.
